# 2003 Annual Conference on Antimicrobial Resistance: Science – Prevention – Control

**DOI:** 10.3201/eid0905.055678

**Published:** 2003-05

## Hyatt Regency Bethesda; Bethesda, Maryland; June 23–25, 2003

The 2003 Conference on Antimicrobial Resistance will be held June 23–25, 2003, in Bethesda, Maryland. In addition to exploring the science, prevention, and control of antimicrobial resistance, participants will define issues and potential solutions to this problem.

The conference is sponsored by the National Foundation for Infectious Diseases (NFID) in collaboration with nine agencies, institutes, and organizations involved in conducting or promoting research, prevention, and control of antimicrobial resistance.

The deadline for online submission of abstracts for oral and poster presentations is April 1, 2003. Registration fee is $350 until May 16. After that date, the fee will be $400.

For additional information, contact NFID, 4733 Bethesda Avenue, Suite 750, Bethesda, MD 20814-5278; telephone: 301-656-0003, extension 12; fax: 301-907-0878; email: **resistance@nfid.org**. Program announcements and forms for abstract submission, registration, and hotel reservations are also available at http://www.nfid.org/conferences/resistance03/

